# MUC16 contributes to the metastasis of pancreatic ductal adenocarcinoma through focal adhesion mediated signaling mechanism

**DOI:** 10.18632/genesandcancer.104

**Published:** 2016-03

**Authors:** Sakthivel Muniyan, Dhanya Haridas, Seema Chugh, Satyanarayana Rachagani, Imayavaramban Lakshmanan, Suprit Gupta, Parthasarathy Seshacharyulu, Lynette M. Smith, Moorthy P. Ponnusamy, Surinder K. Batra

**Affiliations:** ^1^ Department of Biochemistry and Molecular Biology, University of Nebraska Medical Center, Omaha, NE, USA; ^2^ Department of Biostatistics, University of Nebraska Medical Center, Omaha, NE, USA; ^3^ Fred and Pamela Buffett Cancer Center, Eppley Institute for Research in Cancer and Allied Diseases, University of Nebraska Medical Center, Omaha, NE, USA; ^4^ Department of Pathology and Microbiology, University of Nebraska Medical Center, Omaha, NE, USA

**Keywords:** MUC16, pancreatic cancer, metastasis, FAK, CRISPR/Cas9

## Abstract

MUC16, a heavily glycosylated type-I transmembrane mucin is overexpressed in several cancers including pancreatic ductal adenocarcinoma (PDAC). Previously, we have shown that MUC16 is significantly overexpressed in human PDAC tissues. However, the functional consequences and its role in PDAC is poorly understood. Here, we show that MUC16 knockdown decreases PDAC cell proliferation, colony formation and migration *in vitro*. Also, MUC16 knockdown decreases the tumor formation and metastasis in orthotopic xenograft mouse model. Mechanistically, immunoprecipitation and immunofluorescence analyses confirms MUC16 interaction with galectin-3 and mesothelin in PDAC cells. Adhesion assay displayed decreased cell attachment of MUC16 knockdown cells with recombinant galectin-1 and galectin-3 protein. Further, CRISPR/Cas9-mediated MUC16 knockout cells show decreased tumor-associated carbohydrate antigens (T and Tn) in PDAC cells. Importantly, carbohydrate antigens were decreased in the region that corresponds to MUC16 and suggests for the decreased MUC16-galectin interactions. Co-immunoprecipitation also revealed a novel interaction between MUC16 and FAK in PDAC cells. Interestingly, we observed decreased expression of mesenchymal and increased expression of epithelial markers in MUC16-silenced cells. Additionally, MUC16 loss showed a decreased FAK-mediated Akt and ERK/MAPK activation. Altogether, these findings suggest that MUC16-focal adhesion signaling may play a critical role in facilitating PDAC growth and metastasis.

## INTRODUCTION

Among several molecules that have been shown to be deregulated in pancreatic ductal adenocarcinoma (PDAC), mucins are extensively studied with respect to their role in PC pathogenesis [1-4]. In a physiological setting, mucins are extensively glycosylated as their primary role is to form a physical barrier and protect the epithelial cells from various insults. Whereas during pathological conditions, mucins are differentially regulated and exhibit altered expression and glycosylation patterns that confer significant oncogenic potential [1, 5-8]. Hence, mucins have been identified as novel targets for diagnostic, prognostic and therapeutic purposes [2].

Mucin MUC16 is a type-I transmembrane protein composed of a heavily glycosylated extracellular N-terminal domain, tandem repeat domain and a C-terminal domain [9]. MUC16 is the gold standard biomarker in diagnosing and monitoring ovarian cancer patients [10]. In addition, overexpression of MUC16 has also been observed in endometrial [11], breast [12] and lung [13] cancers. Recently, we and others have shown that MUC16 is overexpressed in PDAC, and the expression increases as cancer progresses from precursor invasive lesions (Pancreatic Intraepithelial Neoplasia (PanIN)) to metastatic PDAC, while it is not detected in the normal pancreas [14-17]. Further, we have also shown that MUC16 expression can be utilized in classifying atypical/suspicious fine needle aspirates as pancreatic adenocarcinoma with 100% specificity [3].

Studies performed to investigate the functional role of MUC16 in ovarian, and breast cancer has implicated MUC16 to play an important role in cell proliferation, resisting apoptosis and immune evasion [12, 18]. Cancer-associated mucin MUC16 is over expressed in PDAC and its expression increases in tandem with PDAC progression [14]. A recent study revealed that binding of MUC16 with mesothelin significantly enhanced PDAC cell motility and invasion through matrix metalloproteinase (MMP)-7 induction [19]. In addition, we have shown that overexpression of MUC16 C-terminal domain promotes nuclear translocation of JAK2, upregulates stemness-specific genes LMO2 and NANOG and thus increases PDAC cell proliferation, motility, and metastasis [20]. The effects of MUC16 on PDAC can be mediated in part by mTOR activation and overexpression of its downstream target c-MYC through dysregulated cellular metabolism and reprogramming [21]. Cell surface MUC16 has been shown to interact with other glycoproteins such as galectins and mesothelin [22-24]. Further, MUC16 has been recently proposed to be a functional ligand to E-and L-selectin [25]. These interactions between the cell surface glycoproteins are proposed to promote adhesion, a critical step in the process of metastasis.

Despite the importance of MUC16 as a biomarker in ovarian cancer and its overexpression levels in various cancers, the biological and mechanistic role of MUC16 in tumor progression and its metastatic ability has not yet been identified in pancreatic cancer. To understand further, we used MUC16 knockdown PDAC cells to determine the oncogenic and metastatic role. Here, we showed that MUC16 knockdown decreases PDAC cell tumorigenesis and metastasis. Mechanistically, MUC16 shows biochemical interaction with mesothelin and galectin-3. Further, our results show that CRISPR/Cas9-based MUC16 knockout cells show decreased tumor-associated carbohydrate antigens (T and Tn) in PDAC cells, which suggests for the decreased lectin binding observed in this study. Our findings also reveal that MUC16 interacts with ERM domain containing protein FAK and suggest the interaction could promote the tumorigenic potential through FAK-mediated Akt and ERK/MAPK signaling. Overall, our results suggest that MUC16 plays an important role in PDAC metastasis; therefore, it may represent a novel target in pancreatic cancer.

## RESULTS

### MUC16 knockdown decreases PDAC cell proliferation

**Figure 1:**
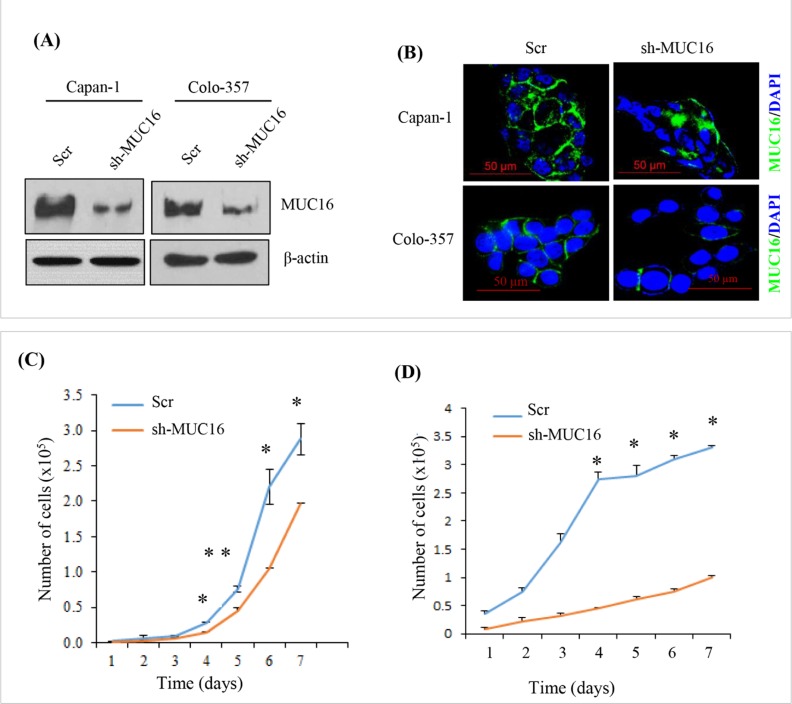
MUC16 knockdown alters in capan-1 and colo-357 PDAC cell proliferation **(A).** Immunoblot analyzes were performed to detect the MUC16 expression in MUC16 knock down capan-1 and colo-357 cell lines. A 40μg of total protein was resolved electrophoretically on a 10% SDS gel, transferred on to PVDF membranes and probed with the anti-MUC16 antibody. β-actin was used as a loading control. **(B).** Immunofluorescent analyzes of MUC16 expression in MUC16 knock down capan-1 and Colo-357 cell lines. MUC16 knockdown colo-357 and capan-1 cells with respective Scr transfected cells were cultured on the coverslip for 48 hours. 48 hours later, cells were fixed and probed with the anti-MUC16 antibody. Primary antibody probing was followed by FITC-conjugated anti-mouse secondary antibody and counterstained with DAPI. All the microscopic pictures are in the similar magnification (x630); scale bar: 50 μm. The MUC16 knock down capan-1 **(C)**, and Colo-357 **(D)** cells were plated in triplicates in six-well plates at a density of 10×10^4^ cells/well and cultured in 2% serum containing media. The cells were trypsinized and counted every 24 hours for seven days, and the growth curve was plotted for the number of cells counted *versus* time of incubation. * indicates *p* < 0.05 and ** indicates *p* < 0.01.

To elucidate the role of MUC16 in PDAC cells, MUC16 was knock down using shRNA in capan-1 and colo-357 PDAC cells. Immunoblot analysis shows a substantial decrease in the expression levels of MUC16 in knockdown cells compared to Scr control (Figure 1A). Similarly, immunofluorescence study also revealed a reduction in the expression of MUC16 in knockdown cells (Figure 1B). To evaluate the functional role of MUC16 in PDAC, we determined the cell growth rate in MUC16 knockdown cells. Compared to the Scr control cells, MUC16 knockdown capan-1 cells show a significant reduction in cell proliferation rate (Figure 1C). Similarly, MUC16 knockdown in Colo-357 cells significantly reduced the total cell number starting from day 1, and the cell growth rate was significant from day 4 until day 7 (Figure 1D).

### MUC16 knockdown decreases colony formation and migration *in vitro*

**Figure 2:**
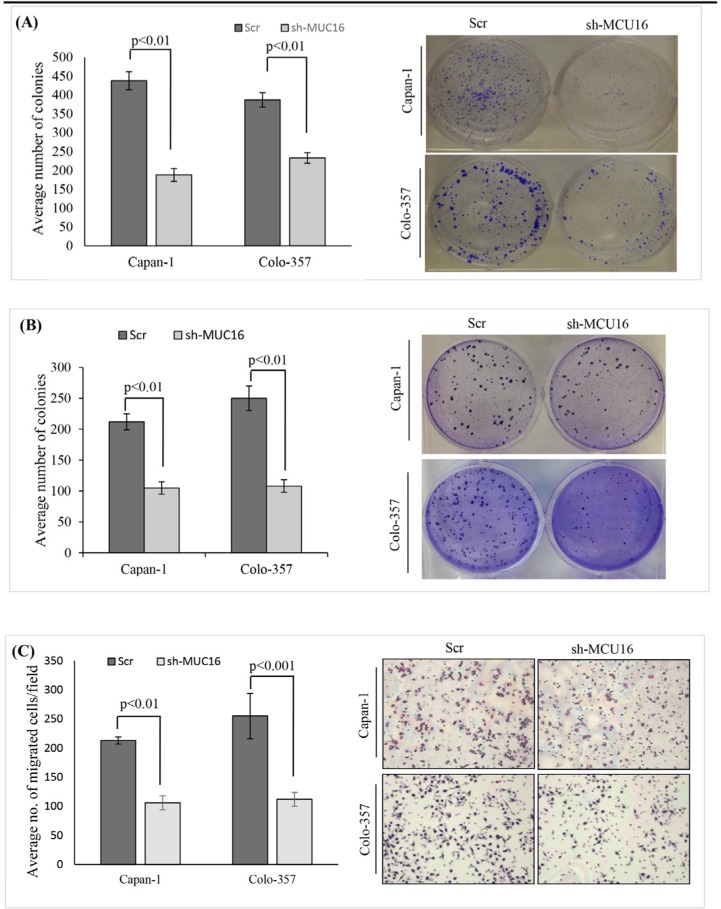
MUC16 expression loss decreases colony formation and migration of PDAC cells **(A).** Colony-forming ability of MUC16 knock downed cells in comparison with respective Scr transfected cells were performed *in vitro*. Both colo-357 and capan-1 cells were plated at a density of 2 × 10^3^ cells per well in a six-well plate. After 16 hours incubation, the unattached cells were removed by changing the media. The cells were fed fresh media once in three days. Twelve days later, the cells were washed with PBS, stained with crystal violet and photographed. The bar diagram on the left indicates the number of colonies. The panel on the right shows the representative picture of respective groups. **(B).** MUC16 knockdown colo-357 and capan-1 with respective Scr control cells were plated at a density of 5 × 10^2^ cells/cm^2^ in soft agar plates. After 24 hours, the dish containing double cells were excluded. Cells were fed with fresh culture media twice a week. By the end of 5 weeks, the colonies formed were stained with 0.1 % crystal violet and counted. The bar diagram on the left indicates the number of colonies. The right panel shows the representative picture of colonies. **(C).** The migratory ability of MUC16 in colo-357 and capan-1 cells. MUC16 knockdown colo-357 and capan-1 with respective Scr control cells of 1×10^6^ cells were plated on top of the 6-well insert. After 24 hours, the cells that migrated were fixed, stained and counted. The data on the left indicates the average number of PDAC cells per field of view (original magnification ×10). The right panel represents the cells from different groups.

To explore the tumorigenic potential of MUC16, *in vitro* clonogenic assay was performed. As shown in Figure 2A, after 10 days of culture, both capan-1 and colo-357 MUC16 knockdown cells displayed a significant (*p* < 0.01) reduction in clonogenic cell survival. We further tested the colony forming efficacy of MUC16 using soft agar assay. As shown, the Scr control cells formed the visible and abundant number of colonies (Figure 2B). However, MUC16 knockdown markedly reduced both the number and size of the colonies in capan-1 and colo-357 cells (*p* < 0.01). To determine the chemotactic impact of MUC16 in capan-1 and colo-357 PDAC cells, we used transwell migration chamber. As expected, increased migration was observed in Scr control cells (Figure 2C). While, the migration was significantly reduced after 24 h in MUC16 knockdown capan-1 (*p* < 0.01) and colo-357 (*p* < 0.001) cells (Figure 2C).

### MUC16 knockdown decreases orthotopic tumor growth and metastasis of PDAC cells

**Figure 3:**
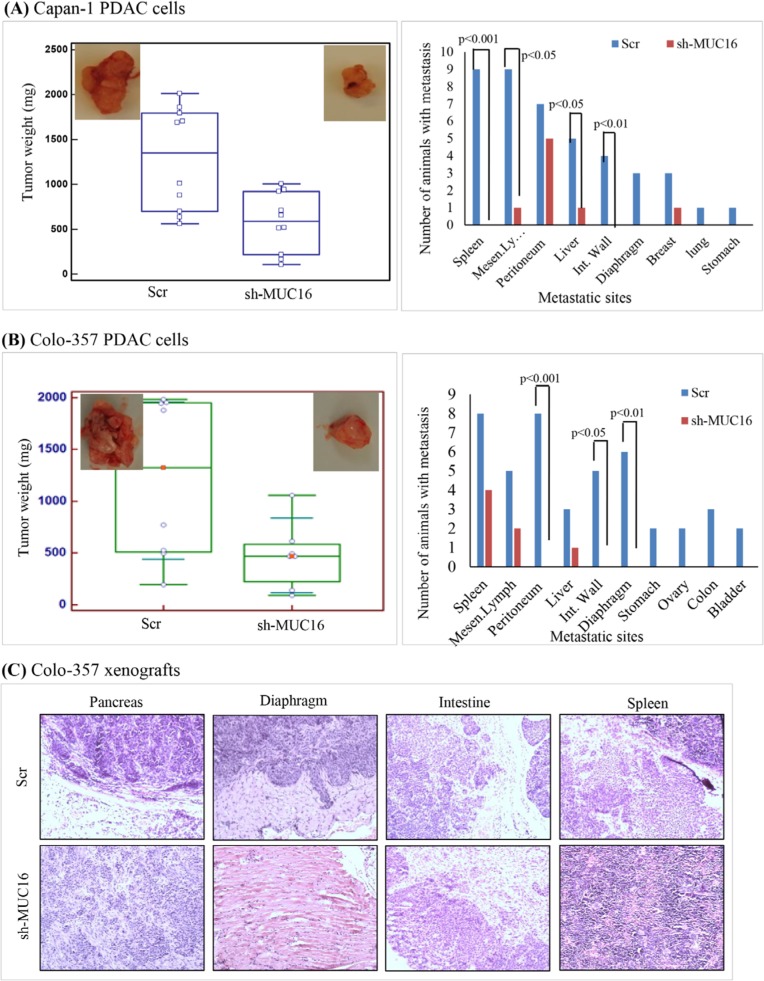
Loss of MUC16 expression decreases the in vivo tumorigenic potential of PDAC cells **(A).** MUC16 depleted (sh-MUC16) and scramble vector (Scr) transfected capan-1 cells in 0.05 ml orthotopically injected into the nude mice. Ten mice were assigned to each group. Mice were continuously monitored for growth and weight. After 21 days, the tumors were resected and weighed. All the major organs were dissected, and metastatic tumor growth was analyzed by both visually and microscopically in H & E staining. Box plot on the left panel indicates the orthotopic tumor growth. The tumor weights of each mouse are represented by a dot. Right panel indicates the number of metastasis incidence from respective group. **(B).** MUC16 depleted (sh-MUC16) and scramble vector (Scr) transfected colo-357 cells were orthotopically injected into the nude mice. Eight and seven mice were assigned to Scr and sh-MUC16 group, respectively. Mice were continuously monitored for growth and weight. After 60 days, the tumors were resected and weighed. All the major organs were dissected, and metastatic tumor growth was analyzed by both visually and microscopically in H & E staining. Box plot on the left panel indicates the orthotopic tumor growth. The tumor weights of each individual mice are represented as a dot. Right panel indicates the number of metastasis incidence from respective group. **(C).** Hematoxylin and eosin stain of xenograft in primary and metastatic sites of colo-357 xenografts in orthotopic model. Colo-357 xenografts were harvested and processed for H and E staining on primary as well as metastatic sites. All the micrographs are in the same magnification (original × 10).

The results obtained from *in vitro* studies suggest that MUC16 contributes to the tumorigenic potential of PDAC cells. To corroborate the *in vitro* findings, we examined whether silencing of MUC16 affects tumor development in nude mice. Orthotopic implantation was carried out using capan-1 and colo-357 PDAC cells. The control capan-1 (Scr) cells developed solid tumors of an average weight of 1400 mg, which confirms the oncogenic potential of MUC16. Whereas, we observed significantly (*p*-value = 0.004) smaller tumors in mice injected with MUC16 knockdown (sh-MUC16) capan-1 cells (Figure 3A, left panel). Further, to analyze the metastatic potential of MUC16, all major organs were dissected and observed for visible metastasis. A significant difference was observed in the metastatic burden in distant organs such as mesenteric lymph node, peritoneum, liver, diaphragm and intestinal wall (Figure 3A; right panel; Table 1) between the MUC16 knockdown and control cells. Similarly, when compared to control, the MUC16 knockdown colo-357 cells show decreased tumor formation in pancreas (Figure 3B, left panel) and metastatic burden (Figure 3B, right panel; Table 1). In addition, splenic adhesions were observed in all the mice that received control (Scr) PDAC cells. H&E staining on both primary and metastatic tissues further validate the tumor growth (Figure 3C).

**Table 1:** Significant tumor burden between colo-357 Scr and sh-MUC16 cells implanted mice (Based on macro metastasis on xenograft animals)

Xenograft tissue site	Colo-357 p-value	Capan-1 p-value
Pancreas	0.043	0.023
Lymph node/Mesentric Lymph node	0.31	0.0011
Omentum/Peritoneum	0.0002	0.65
Spleen	0.077	0.0001
Int. Wall	0.026	0.087
Diaphragm	0.007	0.21
Liver	0.57	0.14
Stomach	0.47	1
Ovary	0.47	
Colon	0.2	
Bladder	0.42	0.58

### MUC16 expression correlates with *in vivo* tumor growth inhibition and decrease in metastasis

**Figure 4:**
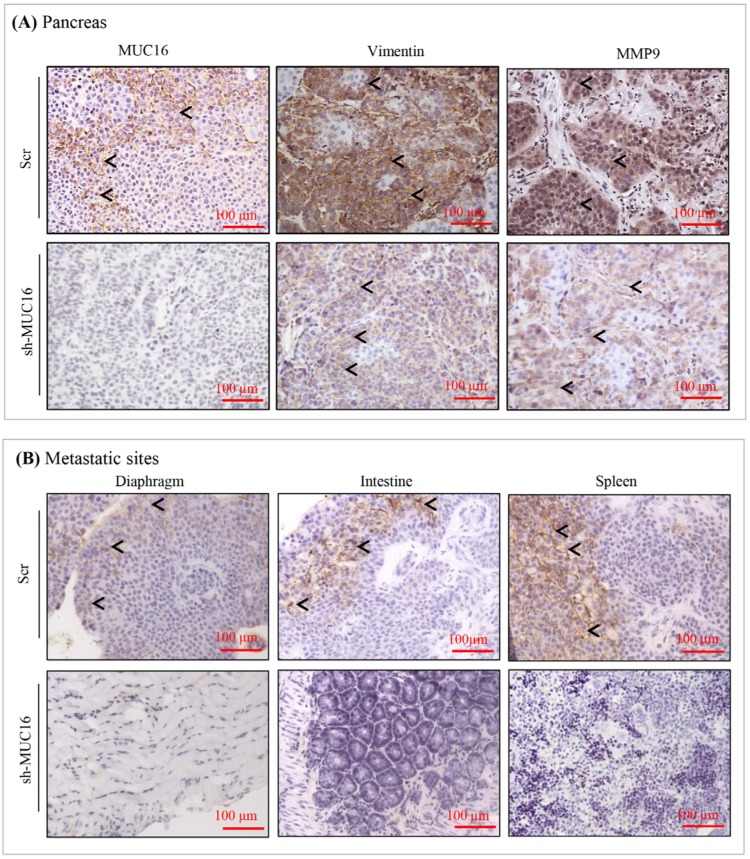
Immunohistochemical analyses of MUC16 and metastatic markers in primary and metastatic sites of colo-357 xenografts **(A).** Immunohistochemical staining for MUC16, vimentin and MMP9 in colo-357 cells implanted pancreas. Black arrow heads indicates the immunostaining of MUC16 (cell surface), vimentin (cytoplasm) and MMP9 (cytoplasm and extracellular). **(B).** Immunohistochemical staining for MUC16 in metastatic site of colo-357 cells implanted mice. Black arrow in the representative image indicates the cell surface immunostaining of MUC16. Nuclei are stained with hematoxylin (blue). All the micrographs are in the same magnification (original × 20).

To confirm the metastatic observations (Figure 3), we performed immunohistochemistry for the expression of MUC16 and other metastatic markers in xenograft tissues. As shown in Figure 4A, cell surface expression of MUC16 is detected in Colo-357 Scr cells, but not in sh-MUC16 cells implanted xenografts. Interestingly, in correlation with MUC16 expression, the expression of vimentin and MMP-9 is substantially higher in colo-357 Scr cells implanted xenografts (Figure 4A). On the other hand, vimentin and MMP-9 expression is reduced in sh-MUC16 xenografts (Figure 4A). Furthermore, in the metastatic xenograft tissue sections, MUC16 staining showed a higher cell surface expression pattern in diaphragm, intestine and spleen (Figure 4B, Top panel), compared with respective control tissues obtained from sh-MUC16 implanted mice. This results suggests that MUC16 depletion results in reduced vimentin and MMP-9 expression, and possibly decrease the metastatic ability of colo-357 cells.

### MUC16 interacts with mesothelin in colo-357 cells and xenograft tissues

**Figure 5:**
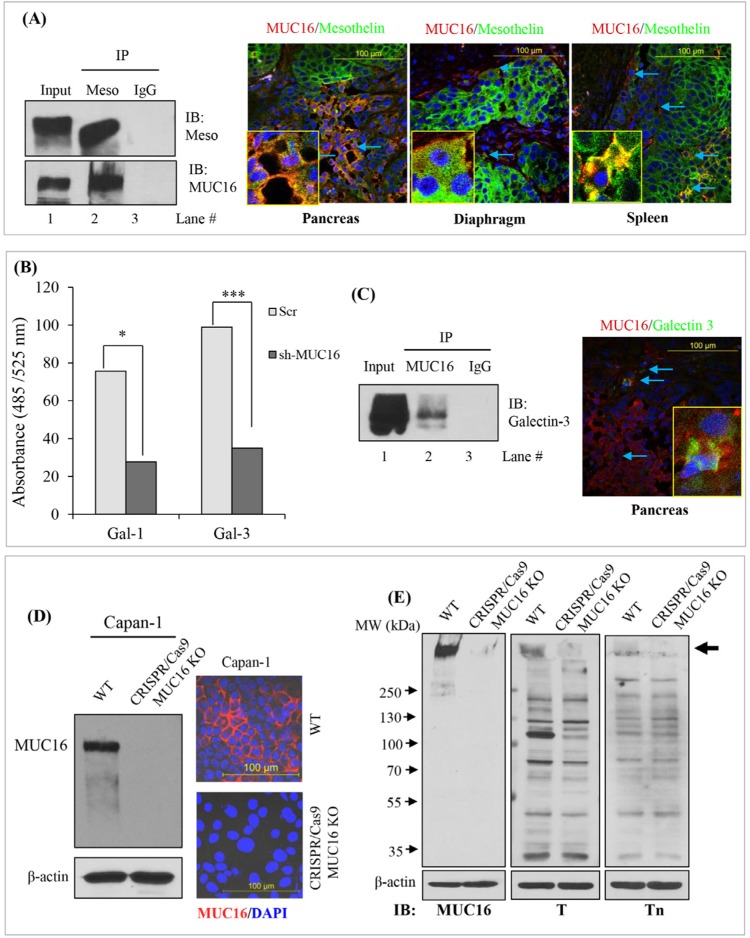
MUC16 interact with mesothelin and galectin-3 in PDAC cells **(A).** Endogenous MUC16 and mesothelin protein interaction in colo-357 cells were analyzed by co-immunoprecipitation and immunoblotting using specific antibodies. IgG was used as the negative control with the same amount of protein (left panel). Right panel shows the MUC16-mesothelin interaction in colo-357 cells implanted mice pancreas and other metastatic sites. Colocalization were enlarged and presented as inset. All the microscopic pictures are in the similar magnification (scale bar: 100 μm). **(B).** MUC16 knockdown colo-357 cells were plated in triplicate to galectin-1 and galectin-3 protein-coated 96-well plates. After 1 h incubation at 37°C, unattached cells were washed carefully with PBS twice. The adhered cells were incubated with Calcein-AM dye, and the fluorescence was measured. **(C).** MUC16 interaction with galectin-3 were determined by immunoprecipitation and Immunofluorescence analyzes. Colo-357 cells were immunoprecipitated using MUC16 antibody and were probed using galectin-3 specific antibodies. IgG was used as the negative control with the same amount of protein (left panel). The right panel shows the MUC16-galectin-3 interaction in primary site of the pancreas. All the microscopic pictures are in the similar magnification (scale bar: 100 μm). **(D).** Immunoblot analyzes were performed to determine the MUC16 expression level in CRISPR/Cas-mediated MUC16 knockout in capan-1 cells. A 40 μg of total protein was resolved in SDS-PAGE, transferred on to PVDF membranes and probed with the anti-MUC16 antibody. β-actin was used as a loading control (left panel). Right panel show immunofluorescent analyzes of MUC16 expression in parental and MUC16-deleted capan-1 cells. Confocal images are in the similar magnification (scale bar: 100 μm). **(E).** Immunoblot analysis were carried out to determine the T and Tn antigen level in wildtype and MUC16 CRISPR/Cas-based knockout capan-1 cells. The immunoblot shows a clear decrease in both T- and Tn-antigen level in MUC16 depleted samples. Solid black arrow indicates the MUC16 migration position in PAGE. MSLN: mesothelin; Gal-1: galectin-1; Gal-3: galectin-3; T: Thomsen-Friedenreich and Tn: Thomsen-nouvelle antigens.

Colo-357 cell lysates were immunoprecipitated with anti-mesothelin antibody and subjected to immunoblot analysis using the MUC16 and mesothelin-specific antibody. As shown in Figure 5A, mesothelin can be precipitated from colo-357 cells. Further, the results indicate that endogenous MUC16 was co-immunoprecipitated by anti-mesothelin (Figure 5A, lane 2, left panel). Next, we investigated the molecular interaction between MUC16 with mesothelin in *in vivo* condition. Immunofluorescence study was performed on colo-357 xenografts. Xenograft tissue sections were processed and probed with a combination of MUC16 with mesothelin antibodies. As shown in Figure 5A (right panel), MUC16 expression is observed in all the tissue sections with detectable cell surface staining. As expected, in the pancreas, MUC16 colocalizes with mesothelin. In metastatic tissue (diaphragm and spleen) sites, as shown in Figure 5A, it is apparent that both MUC16 and mesothelin colocalizes as seen by membranous for MUC16 and membranous with cytoplasmic staining for mesothelin. However, fluorescence microscopy revealed comparatively lesser expression level of MUC16 in diaphragm. The colocalization were shown as inset. These results are consistent with the observed physical interaction of MUC16 with mesothelin (Figure 5A, left panel).

### MUC16 knockdown decreases cell adhesion property of colo-357 cells

It is established that cell surface glycans such as mucins and integrins can interact with β-galactoside-binding lectins such as galectin-3 for forming molecular lattices [26, 27] and plays key roles in the extracellular modulation of tumor progression [28, 29]. To examine whether MUC16 interacts with s-type lectins (disulphide bond dependent attachment) such as galectins in PDAC, MUC16 knockdown colo-357 cells were incubated in galectin-1 and galectin-3 coated plates for an hour and stained with calcein AM dye. Fluorescent absorbance revealed that MUC16 knockdown cells show significant decrease in binding to both galectin-1 and galectin-3 recombinant proteins. Interestingly, colo-357 cells show higher binding affinity towards galectin-3 than galectin-1 (Figure 5B).

### MUC16 interacts with galectin-3 in colo-357 cells and xenograft tissues

Next, we investigated whether MUC16 precipitates galectin-3. Colo-357 cell lysates were immunoprecipitated with anti-MUC16 antibody and subjected to immunoblot analysis using the galectin-3 specific antibody. The results, as indicated in Figure 5C shows an interaction between MUC16 and galectin-3 protein (Figure 5C; lane 2). To investigate the molecular interaction between MUC16 with with galectin-3 in *in vivo* condition, immunofluorescence study was performed on colo-357 xenografts. Xenograft tissue sections were processed and probed with a combination of MUC16 with galectin-3 antibodies. As expected, in the pancreatic tumor, MUC16 colocalizes with galectin-3 (Figure 5C). However, fluorescence microscopy revealed comparatively lesser expression level of galectin-3 in the pancreatic tumor. These results are consistent with the observed physical interaction of MUC16 with galectin-3 (Figure 5C; left *vs*. right panel).

### Generation and validation of CRISPR/Cas9-based MUC16 knockout cell lines

MUC16 deletion in capan-1 cells were carried out by CRISPR/Cas9 system [30]. Briefly, capan-1 PDAC cells were transfected with MUC16 guideRNA (5′-AACACACTCGATGGCGACTC-3′, targeting DNA within the third exon) containing CRISPR/Cas9 vector (pSpCas9 BB-2A-GFP PX458) (Genescript, Piscataway, NJ, USA). 72 hrs later, GFP positive cells were isolated and single cells were captured in a 96-well plate by FACS. Cells were allowed to grow in to colonies. Clones were then expanded and screened for the expression of MUC16 using western blot and confocal microscopic analyses. The absence of MUC16 protein expression in capan-1 cells was confirmed by western blot analysis and confocal microscopy studies (Figure 5D). Immunoblot analysis shows a complete absence of MUC16 expression levels in capan-1 knockout cells compared to untrasfected parental control cells (Figure 5D, left panel). Similarly, immunofluorescence study also revealed the absence of cell surface expression of MUC16 in knockout cells (Figure 5D, right panel). Western blot analysis and the confocal microscopy studies (Figure 5D) togethter show the complete deletion of MUC16 in capan-1 cells.

### MUC16 knockout is associated with decreased expression of Thomsen-Friedenreich (TF/T) and Thomsen-nouvelle (Tn) carbohydrate antigens in capan-1 cells

Aberrant glycosylation of mucins have been shown to facilitate maliganant transformation of pancreatic cancer cells through cell-cell interactions [5, 31]. It has been shown that the tumor-associated carbohydrate antigens such as T and Tn are widely distributed in the tandem repeat region of high molecular weight mucins [5, 31, 32]. Further, binding of these carbohydrate antigens to bound and circulating galectins has been implicated in metastasis. Since, we observed decreased binding of MUC16-knockdown cells to galectin coated plates (Figure 5B), we wanted to investigate whether the decrease in galectin binding is due to decreased expression of MUC16 associated carbohydrate antigens such as Tn and T, which are the preferred ligands for galectin receptors. Lectin blot analysis of MUC16 knockout PDAC cells revealed significant reduction in Tn and T carbohydrate antigens as compared to untransfected control cells (Figure 5E). Interestingly, the decrease was observed in region that corresponds to MUC16, which indicates that decreased binding to galectins might be attributed to decrease in glycans associated with MUC16.

### Novel interaction of MUC16 and ERM domain containing protein FAK in PDAC cells

**Figure 6:**
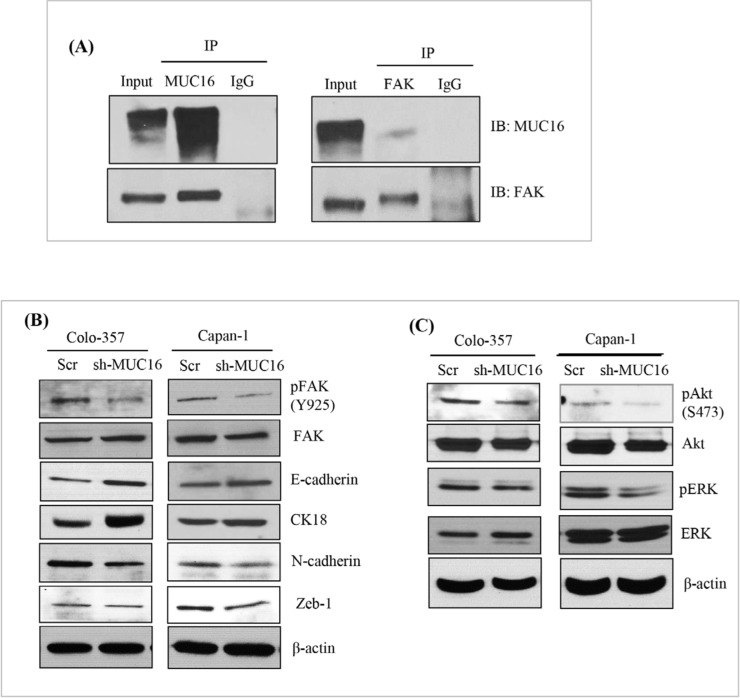
Novel interaction of MUC16 with FAK and activates its downstream signaling in PDAC cells **(A).** Colo-357 cell lysates were pull down using either MUC16 or FAK or respective IgG antibodies. Immunoblot of bound fractions confirms the protein of interest expression. Reciprocally the MCU16 pulldown samples were probed with anti-FAK antibodies and vice versa. IgG was used as a negative control with the same amount of protein. **(B).** MUC16 knockdown colo-357 and capan-1 with respective vector control cells were cultured for 48 hours. After 48 hours, MUC16 knockdown with respective vector control cells were collected, lysed and analyzed for p-FAK (Tyr925), FAK, E-cadherin, CK18, N-cadherin, and Zeb-1 protein levels. β-actin protein level was used as loading control. **(C).** MUC16 shRNA and vector transfected colo-357 and capan-1 cells were maintained under the same condition as described above. The lysates were analyzed for FAK-mediated AKT and ERK/MAPK phosphorylation using immunoblotting analyzes. β-actin protein level was used as loading control.

As it is well established, MUC16 is composed of N-terminal, tandem repeat, and a C-terminal domains. The C-terminal domain further consists of an extracellular portion, transmembrane domain (TM) and a cytoplasmic tail. The MUC16 cytoplasmic tail has an ERM motif which is composed of polybasic sequence of amino acids (RRRKK) for potential interaction with the actin cytoskeleton [9]. This polybasic sequence motif is predicted to bind to a class of ezrin/radixin/moesin (ERM) family of proteins [33]. The ERM proteins can interact with numerous membrane-associated proteins and the actin cytoskeleton for modulating cell structure and play a role in EMT process. To determine if there is any interaction between MUC16 and FAK, an ERM domain containing protein in PDAC cells, in the present study, immunoprecipitation was performed. Colo-357 cells were immunoprecipitated with either mouse MUC16 or rabbit FAK antibody. As shown in Figure 6A, endogenous MUC16 and FAK was immunoprecipitated by respective antibodies, but not by control IgG antibodies (Figure 6A). In a reciprocal approach, the antibody pulldown lysates from colo-357 cells were probed with other sets of antibodies. As observed, our results show the novel interaction between MUC16 and FAK in a physiological setting in PDAC cells.

### MUC16 knockdown decreases FAK-mediated Akt and MAPK activation

Our experimental data reveal that there is an physical association of MUC16 with FAK, which suggests that FAK could be involved in MUC16-mediated metastasis and signaling. In addition, *in vivo* studies showed that mice injected with MUC16 knock down capan-1 and colo-357 cells had decreased metastasis (Figure 3). Since EMT is an important underlying phenomenon for metastasis, we evaluated the expression of EMT markers such as E-cadherin, N-cadherin, cytokeratin-18 and the transcription factor such as ZEB1. As expected, when compared to the control, phosphorylation of FAK but not total FAK protein expression level is reduced in both capan-1 and colo-357 MUC16 knockdown cells (Figure 6B). Further, MUC16 knockdown cells show decreased expression levels of N-cadherin and ZEB-1. While epithelial cell-specific markers such as E-cadherin and cytokeratin-18 is markedly increased in MUC16 knockdown cells (Figure 6B). The phosphorylation of FAK can activate its downstream MAPK or Akt signaling for the migratory property or their survival. Therefore, to investigate the precise underlying downstream signaling in MUC16 knockdown cells, we examined the MAPK and Akt signaling proteins using immunoblot analysis. As shown in Figure 6C, compared to the vector control cells, MUC16 knockdown cells were found to have reduced levels of phospho-Akt and phospho-p42/p44/MAPK. Similarly, in CRISPR/Cas9-based MUC16 knockout capan-1 cells, MUC16 deletion correlates with decreased FAK phosphorylation, N-cadherin and ZO-1 (Supp. Figure 1). Whereas epithelial cell-specific marker E-cadherin is markedly increased in MUC16 deleted capan-1 cells (Supp. Figure 1). Collectively, these results provide compelling evidence that MUC16 depletion leads to decreased focal adhesion and mesenchymal markers and thus contributes to the metastatic behavior of PDAC cells.

## DISCUSSION

The salient feature of the present study is the identification of MUC16 role in PDAC tumorigenesis and metastasis. First, we have shown that MUC16 knockdown significantly decreases PDAC cell proliferation and motility. Second, the results indicate that MUC16 knockdown is involved in the reduction of tumorigenesis and metastasis. Third, a novel interaction of MUC16 was observed with FAK in addition to MUC16 with Mesothelin and Galectin-3. Fourth, we have shown that CRISPR/Cas9-based MUC16 knockout is associated with decrease in carbohydrate antigens (T and Tn) and mechanically suggests the reason for decreased MUC16-galectin interactions. Finally, of the downstream effectors, our results suggest that MUC16 and FAK interaction can activate Akt and ERK/MAPK signaling to promote tumorigenesis and metastasis of PDAC cells. To best of our knowledge, this is the first study reporting the association of MUC16 interaction with FAK with increased aggressiveness of PDAC cells.

Silencing of MUC16 showed a significant decrease in the proliferation of PDAC cells. Recently, we have shown that MUC16 knockdown in breast cancer cells resulted in the rapid transition from G1/S phase and subsequent arrest in the G2/M phase [12]. Further, it has also been shown that knock down of MUC16 in breast cancer cells resulted in a decrease of Cyclin B1 and activation of Aurora Kinase A levels. Since both the molecules play an important role in facilitating cell cycle progression, specifically the G2/M transition, it was inferred that MUC16 enabled rapid cell proliferation *via* cyclin B1 and aurora kinase A [34-36]. Hence, we speculate that MUC16 might be performing a similar function during PDAC cell proliferation as aurora kinase A is elevated in PDAC cells [37].

MUC16 knockdown cells show a significant reduction in tumor formation and metastasis in immunocompromised mice. Notably, mice bearing tumors of MUC16 knockdown cells has a reduction in tumor weight (Figure 3 and Supp. Figure 2) and number of metastasis (Supp table 1). In addition, we observed that MUC16 knockdown completely eliminated the metastatic tumor growth in diaphragm and intestinal wall, and also reduced the metastatic burden in spleen and peritoneum (Figure 3). To further confirm the effect of MUC16 on metastasis, we examined the expression of MUC16 and other metastatic markers in colo-357 xenografts. A significant amount of cell surface staining for MUC16 was observed in Scr xenografts compared with undetectable level in sh-MUC16 xenografts (Figure 4). Correspondingly, notable vimentin and MMP-9 expression was observed in Scr compared with sh-MUC16 xenografts (Figure 4A). These results together with the difference in macroscopic metastasis and *in vitro* colony formation and migration suggested that MUC16 plays an vital role in PDAC metastasis. Our data are consistent with our recent findings that overexpression of MUC16 C-terminal domain resulted in significantly higher tumor growth and metastatic burden towards diaphragm and peritoneum [20]. These data are supported by the hypothesis that organ-specific host microenvironment may play a pivotal role in determining the tumor growth [38]. Further, it has also been observed that with similar tumor size, the number of metastasis to other internal organs is reduced in MUC16 knockdown cells (Supp Table 1). These results suggest that the decrease in size and the number of metastases are not only due to the slower tumor cell proliferation as evidenced in MUC16 knockdown cells (Figure 1), but also due to reduced cancer cell invasiveness (Figure 2).

Recent evidence indicate that MUC16 has been crucial in facilitating metastasis of ovarian cancer cells to peritoneum *via* its interaction with mesothelin [39, 40]. In addition, it has been shown that MUC16 interacts with mesothelin and promotes PDAC cell motility and invasion through MMP-7 activation [19]. Previous results also suggest that MUC16 interaction with lectins can enhance the metastatic potential of PDAC cells [16, 41]. To determine whether similar interactions are observed in our study, we performed immunoprecipitation and colocalization studies. Our results confirm that both MUC16 and mesothelin shows physical interaction in PDAC cells. In addition, MUC16 knockdown cells shows decreased cell adhesion in galectin-1 and -3 coated plates. Truncated carbohydrate antigens present on mucin glycoproteins have been shown to facilitate metastasis of pancreatic cancer cells through cell-cell interactions. Our result (Figure 5E) suggests possible link of aberrant glycans on MUC16 in MUC16-galectin-3 mediated metastasis. Thus, from this study we have shown that MUC16 contributes towards the metastatic function of PDAC cells.

The membrane-bound mucin, MUC16 has an N-terminal domain, tandem repeat and C-terminal domain containing a short transmembrane domain and a cytoplasmic tail containing ERM domain binding motif. These FERM-binding regions may interact with FERM domain containing proteins. FAK belongs to non-receptor protein tyrosine kinase family has an N-terminal containing FERM domain, a central kinase domain and a carboxyl-terminal domain [42]. This FERM domain of FAK can facilitate association with other partner proteins such as integrins and actin cytoskeletons. The FERM domain containing ERM proteins can interact directly with the cytoplasmic regions of transmembrane receptors [43-45] and can coordinate diverse cellular responses, including cell adhesion, polarization, migration, survival, and death [46]. Our recent observation also reveals that overexpression of C-terminal MUC16 increases PDAC proliferation, motility, and metastasis [20]. Hence, MUC16 can bind to the FERM domain of FAK and can activate FAK at focal adhesions. To better understand the MUC16 interaction within the normal physiological conditions, we performed MUC16-FAK interaction. From this study, we provide the first experimental evidence that MUC16 can interact with FAK and these interactions may play an important role in the cytoskeletal proteins rearrangement for metastatic behavior of the cells. Supportively, in a recent study, it has also been shown that a genetically evolved MUC16 related gene agrin has been shown to activate the integrin-FAK pathway and thereby mediate EMT in hepatocellular carcinoma [47]. However, it remains to be determined whether MUC16-FAK interaction is mediated through the ERM motif of the MUC16 cytoplasmic tail.

Although MUC16 is similar to MUC1 and MUC4 in possessing heavily glycosylated N-terminal domain, SEA domain, transmembrane domain and cytoplasmic tail, it is suggested that MUC16 has several distinctive features in its functions and signaling, and the functional role may vary from other mucins [48]. Evolutionary studies also exposed that MUC16 is evolved from different ancestor proteoglycan, agrin [49]. The present study provides a cue to the underlying molecular mechanism of MUC16 in PDAC. First, it revealed that MUC16 can interact with FAK. Since FAK is known to control the mesenchymal characteristics imparting adhesion and invasiveness in cancer cells [50]. The present results of decreased FAK activation, N-cadherin, and ZEB-1, EMT-related transcription factor protein levels supports our hypothesis that MUC16 knockdown cells can abrogate FAK-mediated invasiveness. We have also shown that protein levels of E-cadherin and cytokeratin 18, well-known epithelial specific markers were increased by MUC16 knockdown cells. Supportively, a recent study also revealed that agrin, MUC16 related gene interact with FAK and agrin-FAK axis drives EMT in hepatocellular carcinoma [47]. Second, MUC16 knockdown decreases FAK activation and FAK-mediated Akt and ERK/MAPK signaling. Thus, MUC16-FAK interaction may probably drive the EMT process in PDAC, corroborated by the evidence in MUC16 knockdown cells. Therefore, MUC16 can mediate a unique link between extracellular surface and FAK signaling critical for regulating tumorigenesis and metastasis.

In summary, in this study we provide the first experimental evidence that MUC16 plays an important role in pancreatic tumor development and facilitates metastasis. Our results together with accumulating evidence about the interaction between tumor-cell glycoprotein such as mucin MUC16 and lectin, galectin-3 for instance in PDAC will pave new ways to interfere with cancer progression. Also, we present a physical relationship between MUC16 and FAK, whereby MUC16 can bring FAK and FAK-mediated cytoskeletal proteins at focal adhesion point for enhancing metastasis. This critical relationship between MUC16 and FAK can facilitate FAK-mediated downstream activation of Akt and ERK/MAPK for the tumor growth and metastasis. Clearly, further studies are required to determine the exact mechanism of action, and whether MUC16-specific targeting of lectins such as siglec, selectins, or galectins were involved in cancer modulation [5, 32]. Given the transmembrane nature of MUC16 and the present demonstration of the new biological role and cellular mechanisms may augment additional therapeutic strategies in the future for the treatment of this deadly cancer.

## MATERIALS AND METHODS

### Cell culture and RNA interference

The human PDAC cell lines used in this study were all obtained from American Type Culture Collection (ATCC, Manassas, VA). The MUC16 shRNA (pSUPER-Retro-MUC16-sh, a kind gift from Dr. Ilene K Gipson, Harvard Medical School) transfection was performed, and the stable knockdown was established in capan-1 and Colo-357 PDAC cells as described [12]. Briefly, MUC16 shRNA and control vectors were transfected into the phoenix packaging cells to produce high titer retrovirus. The viral supernatant were then used to infect the cells, and the selection was performed using puromycin (4 μg/ml) containing medium. The antibiotic-resistant cells were then regularly maintained in puromycin (2 μg/ml) containing DMEM media supplemented with 10% FBS and 1% Penicillin-Streptomycin.

### Growth kinetics

Cell proliferation was performed over a period of seven days. The non-targeting vector control and MUC16 knock down cells were plated in triplicates (1×10^4^ cells) and were cultured in DMEM medium containing 2% FBS. The total number of live cells were counted using the Beckman Coulter Vi-CELLTM on each of the seven successive days.

### Trans-well migration assay

Trans-well migration assays were performed using MUC16 knockdown and respective non-targeting scramble control cells. Approximately 2×10^6^ of capan-1 cells were suspended in serum-free DMEM and were cultured in the upper chamber of polyethylene terephthalate (PET; Becton Dickinson) membranes (pore size, 8 μm; six-well insert) for 24 hrs. For colo-357 cells, approximately 1 × 10^6^ cells were cultured in the upper chamber for 24 h. The bottom of each well is supplemented with DMEM containing 10% FBS. After 24 h, cells that had failed to migrate were gently removed with a cotton swab and the cells that migrated were stained with Diff-Quick cell staining kit (Dade Behring, Inc.). The migrated cells were quantified in 10 different random fields taken by the light microscope at the same magnification.

### Orthotopic tumor implantation

To determine the effect of MUC16 knockdown on tumorigenic and metastatic properties of PDAC cells, orthotopic implantation was performed. Four to six week old, immunodeficient mice (as indicated in figure legends) were purchased and maintained as described [51]. Sub-confluent MUC16 knockdown and non-targeting vector control cells were harvested using trypsin-EDTA and washed once with PBS. Cell viability and the number was determined using the Beckman Coulter Vi-CELLTM instrument and cells were dissolved in PBS at 0.5×10^6^ and 0.25×10^6^ of capan-1 and colo-357 cells, respectively. Single cell suspensions of >90% viability were used for implantation. Animals were anesthetized with the intra-peritoneal injection of ketamine and xylazine mixture (4:1). Cells were then injected into the head of the pancreas using a 30-gauge needle. The wound was then closed with catgut and wound clips. The animals were monitored twice weekly and upon observing a palpable mass, the animals were euthanized by CO_2_ asphyxiation at Day 21 and Day 60 for capan-1 and colo-357 cells, respectively. The pancreas was resected and weighed. All the major organs were resected and observed for any metastasis.

### Confocal microscopy analysis

Capan-1 and colo-357 cells were cultured on cover slips for 48 h and incubated with 0.1 M Hanks buffer for 15 min. Cells were fixed with ice-cold 100% methanol, and non-specific blocking was performed using 10% goat serum, separated by 3×5 min PBS wash. The cells were then incubated overnight with anti-MUC16. The cells were washed with PBS (4×10 min) and incubated with alexa flour-conjugated anti-mouse secondary antibodies (1:300) (Jackson Immunoresearch Labs, Inc.) for 30 min at room temperature. Cells were washed again and mounted on glass slides with anti-fade Vectashield mounting medium containing DAPI [4′, 6-Diamidino-2-Phenylindole, Dihydrochloride] (Vector Laboratories, Burlingame, CA, USA). Laser confocal microscopy was performed using LSM 710 microscope (Carl Zeiss GmbH, Jena, Germany). For immunohistofluorescence analyzes on xenograft tissues, the tissue sections were deparaffinized, rehydrated and antigen retrieval was performed using citrate buffer. Nonspecific blocking with 2.5% horse serum and were then followed by overnight incubation with a combination of primary antibodies at 4°C. After brief washing in PBS, the xenograft tissue sections were incubated with Alexa flour conjugated secondary antibodies and processed for confocal microscopy.

### Immunohistochemistry analyzes of xenograft tissues

The xenografts of the pancreas (primary tumors) and metastatic tissues were dissected and fixed in 10% formalin (Fisher Scientific, Fair Lawn, NJ, USA). The tissues were then embedded in paraffin, and serial tissue sections were made at five μm thickness. Immunohistochemistry were performed as described [52, 53]. Briefly, the sections were deparaffinized and rehydrated gradually. Antigen retrieval was performed using citrate buffer (pH 6.0). Endogenous peroxidase activity was then blocked using 3% H2O2. Nonspecific blocking was done by 2.5% horse serum then followed by primary antibodies (MUC16, 1:100; MMP9, 1:500; Vimentin 1:100) overnight at 4°C. After 1 h with an HRP-labelled secondary antibody incubation, the staining was performed with a diaminobenzidine-positive chromogen (Vector Laboratories, Burlingame, CA, USA). The sections were washed at least 3×5 min with PBS between all subsequent steps. After counterstaining with hamatoxylin, sections were dehydrated using graded alcohol. The tissue sections were incubated with xylene for three 5 min before drying and mounting.

### Galectin binding assay

Galectin binding assay was performed as described [31]. Briefly, MUC16 knockdown colo-357 cells were plated in triplicate in galectin-1 and galectin-3 protein-coated 96-well plates. After 1 h incubation at 37°C, unattached cells were washed carefully with PBS twice. The adhered cells were incubated with Calcein-AM dye for 1 h at 37°C. The relative fluorescence level was measured at an excitation and emission wavelength of 485 and 520 nm, respectively. *p* values of < 0.05 were considered statistically significant.

### Immunoprecipitation

Colo-357 cells were cultured for 48 h, harvested and washed with ice-cold phosphate-buffered saline (PBS), pH 7.0, lysed by pass-through the 27G needle using an immunoprecipitation (IP) buffer containing 50 mM Tris-HCl, pH 7.4, 150 mM NaCl, 5 mM EDTA, 0.5% NP-40. Phosphatase and protease inhibitors were added fresh. Three μg of antibodies to the protein of interest or IgG were added to 500 μg of protein lysates and incubated overnight at 4°C in 500 μl of total volume. Antigen-antibody complex was incubated with Protein A/G-Sepharose beads for additional 2 h at 4°C. Beads were then washed at least three times with IP buffer. Immunoprecipitated proteins were eluted by heating at 95°C for 5 min in 2X sample buffer (125 mM Tris-HCl, pH 6.8, 2% SDS (v/v), 0.001% bromophenol blue, 10% glycerol (v/v), 100 mM dithioerythritol) and subjected to immunoblot analyzes. For reciprocal analysis, lysates were precipitated with other sets of antibodies and immunocomplex were analyzed by immunoblot.

### Immunoblot studies

Immunoblot analysis was performed as described [51]. Briefly, protein was extracted from Scr and sh-MUC16 transfected PDAC cells using ice-cold RIPA (50mM Tris-HCl, 150mM NaCl, 1% NP-40, 0.5% sodium deoxycholate and 0.1% SDS) containing protease (1mM phenyl-methyl sulphonyl fluoride, 1mg/ml aprotinin, 1mg/ml leupeptin) and phosphatase inhibitors. Equal amount of proteins were resolved using SDS-polyacrylamide (8-10%) or SDS-agarose gels and then transferred to PVDF membrane. The membranes were then blocked, probed with primary antibodies for overnight at 4°C. After, secondary antibody incubation for an hour, the protein of interest were visualized by an enhanced chemiluminescence detection (Thermo Fisher Scientific, Waltham, MA, USA). For detecting total proteins or loading control, the membranes were stripped and re-probed with antibodies against respective total proteins or β-actin.

### Statistical analysis

Statistical analysis was performed by using Medcalc for Windows version 9.6.4.0 software (MedCalc Software bvba, Mariakerke, Belgium). For xenograft experiments, tumor weight was compared between the groups using Wilcoxon rank sum test. Fisher's exact test was used to compare the proportion of animals those developed metastatic sites between groups, looking at each site separately (affected yes *vs*. no). For all other simple statistical comparison between two groups, Student's t-test were used. A *p*-value of < 0.05 was considered as statistically significant.

## SUPPLEMENTARY MATERIALS FIGURES AND TABLE


