# Supplementary material for: MUC16 contributes to the metastasis of pancreatic ductal adenocarcinoma through focal adhesion mediated signaling mechanism
Source: Genes Cancer. 2016 Mar;7(3-4):110–24. doi: 10.18632/genesandcancer.104 (PMC4918949; doi:10.18632/genesandcancer.104)
Supplement: Supplementary file 1 [file ganc-07-110-s001.pdf]

MUC16 contributes to the metastasis of pancreatic ductal adenocarcinoma through focal adhesion mediated signaling mechanism

Materials and Methods

Subcutaneous tumor implantation

The tumorigenic ability of MUC16 knockdown capan-1 cells was carried out in immunodeficient nude mice (NCI-Fredrick Cancer Research and Development Center, Frederick, MD). The mice were housed under specific pathogen-free conditions and fed sterile water and ad libitum. These mice were treated in accordance with the Institutional Animal Care and Use Committee guidelines. Sub-confluent MUC16 knockdown and non-targeting Scr control capan-1 cells were harvested and washed once with PBS. Cell viability and the number was determined using the Beckman Coulter Vi-CELLTM instrument and cells were made up in PBS at a concentration of 2×10^6 cells/25µl. Single cell suspensions of >90% viability were used for injections. The cells were injected subcutaneously in 10 immunodeficient mice (5 per group). The animals were monitored weekly twice for any tumor growth. The mice were maintained until the observance of palpable mass at the inoculation site. All mice were sacrificed, and the incidence of the tumor was determined.

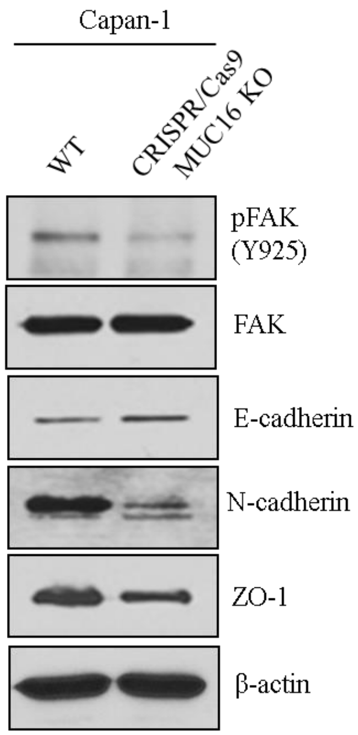

Supp. Figure 1: Effect of CRISPR/Cas-mediated MUC16 knockout on EMT markers in capan-1 PDAC cells. MUC16 knockout and parental capan-1 cells were cultured for 48 hours. Then, cells were lysed and analyzed for p-FAK(Tyr925), FAK, E-cadherin, N-cadherin, and ZO-1 protein levels. β-actin protein level was used as loading control.

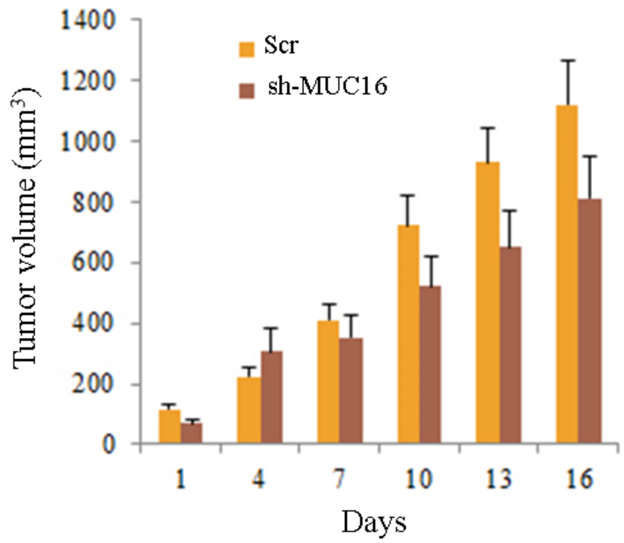

Supp. Figure 2: Loss of MUC16 expression decreases subcutaneous tumor formation in SCID mice. MUC16 shRNA and Scr transfected capan-1 cells in 0.05 ml injected into nude mice per group. After the palpable mass had observed, the tumor size was monitored every other day up to 45 days after inoculation. The mice were euthanized after 45 days, and the pancreatic tumor that had developed was resected. The box plot represents the mean tumor volume.

Supp. Table 1: Metastatic incidence in colo-357 Scr and sh-MUC16 cell xenografts (Based on macro metastasis).

**Colo-357 Xenograft**	**Scr**		**sh-MUC16**	
**Animal No.**	**Tumor weight (mg)**	**Metastatic sites**	**Tumor weight (mg)**	**Metastatic sites**
1	2179	S,P,L,K,I,D	495	
2	1955	S,P,I,D,M	1058	S,L,M
3	497	S,P,L,D	90	
4	771	S,P	468	
5	524.9	S,P,M	613	S,M
6	1948	S,P,L,I,D,M,U	140	S
7	194	S,P,K,I,D,M,C	469	S
8	1984	S,P,I,D,M,U,O,C		

Metastatic sites: S, Spleen; P, Peritoneum; L, Liver; K, Kidney; I, Intestinal wall; D, Diaphragm; M, Mesenteric lymph node; C, Colon; U, Uterus; O, Ovary.
